# Supplementary material for: The Evolution and Characterization of the RNA Interference Pathways in Lophotrochozoa
Source: Genome Biol Evol. 2024 May 7;16(5):evae098. doi: 10.1093/gbe/evae098 (PMC11114477; doi:10.1093/gbe/evae098)
Supplement: evae098_Supplementary_Data [file evae098_supplementary_data.zip › Supplementary_materials.zip!/selected_miRNAs/NoGe_selected_miRNAs/mirdeep_pdf_files/NMRB01000380.1_Notospermus_geniculatus_strain_Ushimado_scaffold380,_whole_genome_shotgun_sequence_11925.pdf]

Provisional ID	:	NMRB01000380.1_Notospermus_geniculatus_strain_Ushimado_scaffold380,_whole_genome_shotgun_sequence_11925
Score total	:	2447.3
Score for star read(s)	:	3.9
Score for read counts	:	2438.6
Score for mfe	:	1.8
Score for randfold	:	0
Score for cons. seed	:	3
Total read count	:	4795
Mature read count	:	4483
Loop read count	:	0
Star read count	:	312

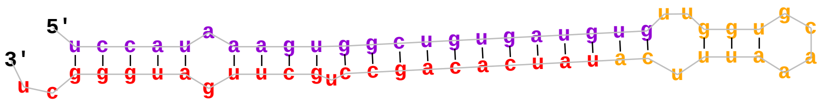

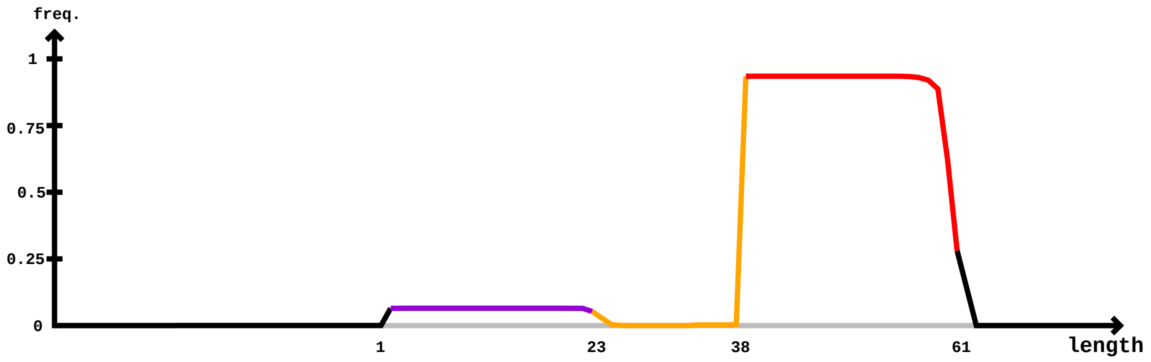


```
                                 Star                                 Mature
5'- ugaauuuuuugcgagugguagucuuccuucuauuccauaaaguggcugugauguguuggugcaaauuucauaucacagccugcuugaugggcuggauggugga accgac   -3'   obs
    ugaauuuuuugcgagugguagucuuccuucuauuccauaaaguggcugugauguguuggugcaaauuucauaucacagccugcuugaugggcuggauggugga accgac         exp
    ...............((((..(((.((.((((.(((((.((((((((((((((((..(((....))).)))))))))))).)))).))))).)))).)).)))))))..   reads   mm   sample
    .................................uccauaNaguggcugugaugug......................................................   1       1    Mm1
    .................................uccauaaaguggcugugaugug......................................................   25      0    Mm1
    .................................................................uuucauaucacagccugcuuga......................   5       0    Mm1
    ......................................................................uaucacagccugcuugaugg...................   12      0    Mm1
    ......................................................................uaucacagccugcuugauggg..................   52      0    Mm1
    ......................................................................uaucacagccAgcuugaugggc.................   1       1    Mm1
    ......................................................................uaucacagccugcuugaugggc.................   69      0    Mm1
    ......................................................................uaucacagccugcuugaCgggc.................   1       1    Mm1
    ......................................................................uaucacagccugcuugaAgggc.................   1       1    Mm1
    ......................................................................uaucacagccugcuugaugAgc.................   2       1    Mm1
    ......................................................................uaucacagccugcuugaGgggcu................   1       1    Mm1
    ......................................................................uaucacagccugcuugaugUgcu................   1       1    Mm1
    ......................................................................uaucacagccugcuugaugAgcu................   4       1    Mm1
    ......................................................................uaucacagccugcuugaugggcA................   5       1    Mm1
    ......................................................................uaucacagccugcuugaugggcu................   87      0    Mm1
    ......................................................................uaucacagccugcuugaAgggcu................   1       1    Mm1
    ......................................................................uaucacagccugcuugaugggcG................   1       1    Mm1
    ......................................................................uaucacagccugcuugCugggcu................   1       1    Mm1

    .................................uccauaaaguggcugugaugu.......................................................   15      0    Mm2
    .................................uccauaaaguAgcugugaugu.......................................................   1       1    Mm2
    .................................uccauaaaguggcugugCugug......................................................   1       1    Mm2
    .................................uccauaaaguggcugugaugug......................................................   35      0    Mm2
    .................................uccauaaaguggcuguCaugug......................................................   1       1    Mm2
    .................................uccauaaaguggcugugaugugu.....................................................   3       0    Mm2
    .................................uccauaaaguggcugugaugugG.....................................................   5       1    Mm2
    .................................uccauaaaguggcugugauguguug...................................................   1       0    Mm2
    ......................................................................uaucacagccugcuugau.....................   1       0    Mm2
    ......................................................................uaucacagccugcuugaug....................   7       0    Mm2
    ......................................................................uaucacagccugcuugauU....................   1       1    Mm2
    ......................................................................uaucacagccugcuugaGg....................   1       1    Mm2
    ......................................................................uaucacagccugcuugaugg...................   11      0    Mm2
    ......................................................................uaucacagccugcuugaugU...................   2       1    Mm2
```

```
                                Star                               Mature
ugaauuuuuugcgagugguagucuuccuucuauuccauaaaguggcugugauguguuggugcaaauuucauaucacagccugcuugaugggcuggaugguggaaccgac
......................................................................uaucacagccugcuugaGggg..................  2    1  Mm2
......................................................................uaucacagccugcuugauggg..................  203  0  Mm2
......................................................................uaucacagccugcuugauggC..................  2    1  Mm2
......................................................................uaucacagccugUuugauggg..................  1    1  Mm2
......................................................................uaucacagccAgcuugauggg..................  1    1  Mm2
......................................................................uaucacagccugcuugUuggg..................  1    1  Mm2
......................................................................uaucacagccugcuugaAggg..................  1    1  Mm2
......................................................................uaucacagccugcuugaugAg..................  1    1  Mm2
......................................................................uaucacagccugcuugaCggg..................  1    1  Mm2
......................................................................uaucacagccugcuugaugUg..................  1    1  Mm2
......................................................................uGucacagccugcuugaugggc.................  1    1  Mm2
......................................................................uaucacagccugcuugaugggU.................  4    1  Mm2
......................................................................uaucacagccugcuugaugAgc.................  6    1  Mm2
......................................................................uaucacagccugcuugauCggc.................  1    1  Mm2
......................................................................uaucacagccugcuugUugggc.................  1    1  Mm2
......................................................................uaucacagccugcuugaAgggc.................  1    1  Mm2
......................................................................uaucacagccugcuugaugggA.................  21   1  Mm2
......................................................................uaucacagccugcuugaugggc.................  353  0  Mm2
......................................................................uaucacagccugcuugCugggc.................  1    1  Mm2
......................................................................uaucacagccugcuCgaugggc.................  1    1  Mm2
......................................................................uaucacagccugcuugaugggG.................  2    1  Mm2
......................................................................uaucacagccugcuugaugggcu................  236  0  Mm2
......................................................................uaucacagccugcuugaugggcG................  7    1  Mm2
......................................................................uaucacagccugcuugaugggAu................  4    1  Mm2
......................................................................uaucacagccugcuugaCgggcu................  1    1  Mm2
......................................................................uUucacagccugcuugaugggcu................  1    1  Mm2
......................................................................uaucacagUcugcuugaugggcu................  1    1  Mm2
......................................................................uaucacagccugcuugaAgggcu................  3    1  Mm2
......................................................................uaucacagccugcuugaugggcA................  2    1  Mm2
......................................................................uaucacagccugcuugGugggcu................  1    1  Mm2
......................................................................uaucacagccugcuugaGgggcu................  1    1  Mm2
......................................................................uaGcacagccugcuugaugggcu................  1    1  Mm2
......................................................................uaucacagccugcuugaugggcuU...............  10   1  Mm2
......................................................................uaucacagccugcuugaugggcug...............  2    0  Mm2

.................................uccauaaaguggcugugaugu.......................................................  6    0  Fm2
.................................uccauaaaguggcugugauguUuu....................................................  9    1  Fm2
.................................................................uuucauaucacagccugcuugau.....................  5    0  Fm2
......................................................................uaucacagccugcuuUau.....................  5    1  Fm2
......................................................................uaucacagccugcuuAaugg...................  1    1  Fm2
......................................................................uaucacagccugcuugaugg...................  20   0  Fm2
......................................................................uaucacagccugcuugauggU..................  1    1  Fm2
......................................................................uaucaAagccugcuugauggg..................  1    1  Fm2
......................................................................uaucacagccugcuugUuggg..................  1    1  Fm2
......................................................................uaucacagccugcuugaAggg..................  1    1  Fm2
......................................................................uaucacagccugcuugauggg..................  96   0  Fm2
......................................................................uaucacagccugcuugaCggg..................  1    1  Fm2
......................................................................uaucacagccugcuugaugggc.................  20   0  Fm2
......................................................................uaucacagccugcuugaugAgc.................  6    1  Fm2
......................................................................uaucacagccugcuugaugggA.................  18   1  Fm2
......................................................................uaucacagccugcuugaugCgcu................  1    1  Fm2

.................................uccauaaaguggcugugaugu.......................................................  1    0  Fm1
.................................uccauaaaguggcugugaugug......................................................  9    0  Fm1
.................................uccauaaaguggcugugauguguu....................................................  4    0  Fm1
..................................ccauaaaguggcugugaugugu.....................................................  1    0  Fm1
.....................................................................auaucacagccugcuuAaugggc.................  1    1  Fm1
.....................................................................auaucacagccugcuugaugggc.................  7    0  Fm1
......................................................................uaucacagccugcuugaug....................  3    0  Fm1
......................................................................uaucacagccugcuugaugg...................  6    0  Fm1
......................................................................uaucacagccugcuugauggg..................  95   0  Fm1
......................................................................uaucacagccugcuugCugggc.................  1    1  Fm1
......................................................................Naucacagccugcuugaugggc.................  1    1  Fm1
......................................................................uaucacagccugcuugaAgggc.................  1    1  Fm1
......................................................................uaucacagccugcuugGugggc.................  1    1  Fm1
......................................................................uaucacagccugcuugaugggc.................  171  0  Fm1
......................................................................uaucacagccugcuugaugggA.................  12   1  Fm1
......................................................................uaucacagccugcuugaGgggc.................  1    1  Fm1
......................................................................uaucacagccugcuugaugAgc.................  7    1  Fm1
......................................................................uaucacagccugcuugaugggU.................  3    1  Fm1
```

```
                                 Star                               Mature
ugaauuuuuugcgagugguagucuuccuucuauuccauaaaguggcugugauguguuggugcaaauuucauaucacagccugcuugaugggcuggaugguggaaccgac
......................................................................uauGacagccugcuugaugggc.................   1   1   Fm1
......................................................................uaucacagccuCcuugaugggc.................   1   1   Fm1
......................................................................uaucacagccugcuugaugggcG................   7   1   Fm1
......................................................................uaucacagccugcuugaugggAu................   1   1   Fm1
......................................................................uaucacagccugcuugaugggcu................   90  0   Fm1
......................................................................uaucacagccugcuugaCgggcu................   1   1   Fm1
......................................................................uaucacagccugcuugaugggcA................   1   1   Fm1
......................................................................uaucacagccugcuugaugggcuU...............   6   1   Fm1

.................................uccauaaaguggcugugau.........................................................   1   0   Mm3
.................................uccauaaaguggcugugauA........................................................   1   1   Mm3
.................................uccauaaaguggcuguAaugu.......................................................   1   1   Mm3
.................................uccauaaaguggcugugaugu.......................................................   7   0   Mm3
.................................uccauaaaguggcugugGugu.......................................................   1   1   Mm3
.................................uccauaaaguggcugugaugug......................................................   36  0   Mm3
.................................uccauaaaguCgcugugaugug......................................................   1   1   Mm3
.................................uccauaaaguggcugugaugugA.....................................................   1   1   Mm3
.................................uccauaaaguggcugugauguUu.....................................................   1   1   Mm3
.................................uccauaaaguggcugugaugugu.....................................................   4   0   Mm3
.................................uccauaaaguggcugugauguguu....................................................   1   0   Mm3
.................................uccauaaaguggcugugauguguuU...................................................   1   1   Mm3
...................................cauaaaguggcugugaugug......................................................   1   0   Mm3
......................................................................uaucacagccugcuugau.....................   1   0   Mm3
......................................................................uaucacagccugcuugaug....................   1   0   Mm3
......................................................................uaucacagccugcuugaugU...................   1   1   Mm3
......................................................................uaucacagccAgcuugaugg...................   1   1   Mm3
......................................................................uaucacagccugcuugaGgg...................   1   1   Mm3
......................................................................uaucacagccugcuugaugg...................   12  0   Mm3
......................................................................uaucacagccugUuugauggg..................   1   1   Mm3
......................................................................uaucacagccuUcuugauggg..................   1   1   Mm3
......................................................................uaucacagccCgcuugauggg..................   1   1   Mm3
......................................................................uaucacagUcugcuugauggg..................   1   1   Mm3
......................................................................uaucacagccugcuugCuggg..................   1   1   Mm3
......................................................................uaucacagccugcuugauggC..................   1   1   Mm3
......................................................................uaucacagccugcuugaAggg..................   1   1   Mm3
......................................................................uaucacagccugcuugauggg..................   116 0   Mm3
......................................................................uaucacagccugcuugaugggG.................   1   1   Mm3
......................................................................uaucacagccugcuugGugggc.................   1   1   Mm3
......................................................................uaucacagccugcuugaugggU.................   2   1   Mm3
......................................................................uaAcacagccugcuugaugggc.................   1   1   Mm3
......................................................................uaucacagccugcuugaugAgc.................   2   1   Mm3
......................................................................uaucacagccugcuugaAgggc.................   1   1   Mm3
......................................................................uaucacGgccugcuugaugggc.................   1   1   Mm3
......................................................................uaucacaUccugcuugaugggc.................   1   1   Mm3
......................................................................uaucacagccugcuugaugggA.................   10  1   Mm3
......................................................................uaucacagccugcuugaCgggc.................   1   1   Mm3
......................................................................uaucacagccCgcuugaugggc.................   1   1   Mm3
......................................................................uaucacagccugcuugaugggc.................   147 0   Mm3
......................................................................uaucacagUcugcuugaugggc.................   1   1   Mm3
......................................................................uaucacagccugcuugaugggcG................   6   1   Mm3
......................................................................uaucacagUcugcuugaugggcu................   2   1   Mm3
......................................................................uaucacagccugcuugaugggcC................   1   1   Mm3
......................................................................uaucacagccugcuugaugggAu................   1   1   Mm3
......................................................................uaucacagccugcuugaGgggcu................   1   1   Mm3
......................................................................uaucacagccugcuugaugggcu................   117 0   Mm3
......................................................................uaucacagcUugcuugaugggcu................   2   1   Mm3
......................................................................uaucacagccugcuugaugggcug...............   1   0   Mm3
......................................................................uaucacagccugcuugaugggcuU...............   1   1   Mm3

...........cgagugguagucGuccuucuau............................................................................   1   1   Fm3
.................................uccauaaaguggcugugaugu.......................................................   24  0   Fm3
.................................uccauaaaguggcugugCugug......................................................   1   1   Fm3
.................................uccauaaaguggcugugUugug......................................................   2   1   Fm3
.................................uccauaaaguggcuguCaugug......................................................   1   1   Fm3
.................................uccauaaaguggcugugGugug......................................................   1   1   Fm3
.................................uccauaaaguggcugugaugug......................................................   83  0   Fm3
.................................uccauaaaguggcugugauguU......................................................   2   1   Fm3
.................................uccauaaaguggcugugaugugu.....................................................   22  0   Fm3
.................................uccauaaaguggcugugaugugA.....................................................   1   1   Fm3
.................................................................uuucauaucacagccugcuugUu.....................   1   1   Fm3
```

```
                                                 Star                                Mature
ugaauuuuuugcgagugguagucuuccuucuauuccauaaaguggcugugauguguuggugcaaauuucauaucacagccugcuugaugggcuggaugguggaaccgac
......................................................................uaucacagccugcuugau.....................  4    0  Fm3
......................................................................uaucacagccAgcuugaug....................  3    1  Fm3
......................................................................uaucacagccugcuugaug....................  33   0  Fm3
......................................................................uaucacagccugcuugaCgg...................  1    1  Fm3
......................................................................uaucacagccugcuugaugg...................  87   0  Fm3
......................................................................uaucacagccugcuugaugU...................  3    1  Fm3
......................................................................uaAcacagccugcuugauggg..................  1    1  Fm3
......................................................................uaucacagccugcuugaCggg..................  5    1  Fm3
......................................................................Caucacagccugcuugauggg..................  1    1  Fm3
......................................................................uUucacagccugcuugauggg..................  1    1  Fm3
......................................................................uGucacagccugcuugauggg..................  2    1  Fm3
......................................................................uaucacagccCgcuugauggg..................  1    1  Fm3
......................................................................uaucacagccugcuugauUgg..................  2    1  Fm3
......................................................................uaucacagcAugcuugauggg..................  1    1  Fm3
......................................................................uaucCcagccugcuugauggg..................  1    1  Fm3
......................................................................uaucacagccugcuugaAggg..................  6    1  Fm3
......................................................................uaucacagcUugcuugauggg..................  9    1  Fm3
......................................................................uaucacagccugcuugCuggg..................  1    1  Fm3
......................................................................uaucacagccugcuugUuggg..................  2    1  Fm3
......................................................................uaucacagccGgcuugauggg..................  1    1  Fm3
......................................................................uaucacagccugcuugaGggg..................  6    1  Fm3
......................................................................uaucacagccugcuugauggg..................  644  0  Fm3
......................................................................uaucacagccugcuugaCgggc.................  4    1  Fm3
......................................................................uaucacagccugcuugaugggU.................  9    1  Fm3
......................................................................uaucacagccugcuugauUggc.................  1    1  Fm3
......................................................................uaucacagccugcuugaugUgc.................  1    1  Fm3
......................................................................uaucacagccAgcuugaugggc.................  3    1  Fm3
......................................................................uaucacagccugcuugCugggc.................  1    1  Fm3
......................................................................uaucacagccugcuugaAgggc.................  1    1  Fm3
......................................................................uaucacagccugcuugauCggc.................  1    1  Fm3
......................................................................uaucacagccugcuugGugggc.................  1    1  Fm3
......................................................................uaucacagccugcuugaugggA.................  96   1  Fm3
......................................................................uaucacagccugcuugaugAgc.................  10   1  Fm3
......................................................................uaucacaUccugcuugaugggc.................  1    1  Fm3
......................................................................uaucacagcUugcuugaugggc.................  4    1  Fm3
......................................................................uaucacagccugcuugaGgggc.................  5    1  Fm3
......................................................................Naucacagccugcuugaugggc.................  1    1  Fm3
......................................................................uaucacagccugcuugUugggc.................  1    1  Fm3
......................................................................uaucacaAccugcuugaugggc.................  2    1  Fm3
......................................................................uaucacagccugcuugaugggc.................  614  0  Fm3
......................................................................uaucacagccuAcuugaugggc.................  1    1  Fm3
......................................................................uaucGcagccugcuugaugggc.................  1    1  Fm3
......................................................................uauAacagccugcuugaugggc.................  1    1  Fm3
......................................................................uauUacagccugcuugaugggcu................  1    1  Fm3
......................................................................uaucacagccugcuugaugggcA................  9    1  Fm3
......................................................................uaucacagccGgcuugaugggcu................  1    1  Fm3
......................................................................uaucacagccugcuugaugAgcu................  1    1  Fm3
......................................................................uaucacagccugcuugaCgggcu................  6    1  Fm3
......................................................................uaucaUagccugcuugaugggcu................  1    1  Fm3
......................................................................uaucacagccugcuugCugggcu................  2    1  Fm3
......................................................................uaucacagccugcuugaAgggcu................  6    1  Fm3
......................................................................uaucacagccugcuuAaugggcu................  1    1  Fm3
......................................................................uaucacagccugcuugaugggAu................  19   1  Fm3
......................................................................uaucacagccugcuugaGgggcu................  2    1  Fm3
......................................................................uaucacagccugcuugaugggcu................  675  0  Fm3
......................................................................uaucacagcUugcuugaugggcu................  7    1  Fm3
......................................................................uaucacagccugcuugaugggcuU...............  8    1  Fm3
```
